# And then there were two

**DOI:** 10.7554/eLife.12724

**Published:** 2015-12-30

**Authors:** Lorraine F Clark, Thomas Kodadek

**Affiliations:** Departments of Chemistry and Cancer Biology, The Scripps Research Institute, Jupiter, United States; Departments of Chemistry and Cancer Biology, The Scripps Research Institute, Jupiter, United Stateskodadek@scripps.edu

**Keywords:** proteome microarray, protein lysine deacetylase, clip-chip strategy, *E. coli*

## Abstract

A second enzyme that removes acetyl groups from lysine residues in *E. coli* been discovered and represents the founding member of a new enzyme family.

**Related research article** Tu S, Guo S-J, Chen C-S, Liu C-X, Jiang H-W, Ge F, Deng J-Y, Zhou Y-M, Czajkowsky D, Li Y, Qi B-R, Ahn Y-H, Cole PA, Zhu H, Tao S-C. 2015. YcgC represents a new protein deacetylase family in prokaryotes. *eLife* **4**:e05322. doi: 10.7554/eLife.05322

**Image** Different lysine deactylases regulate distinct sets of substrates in *E. coli*

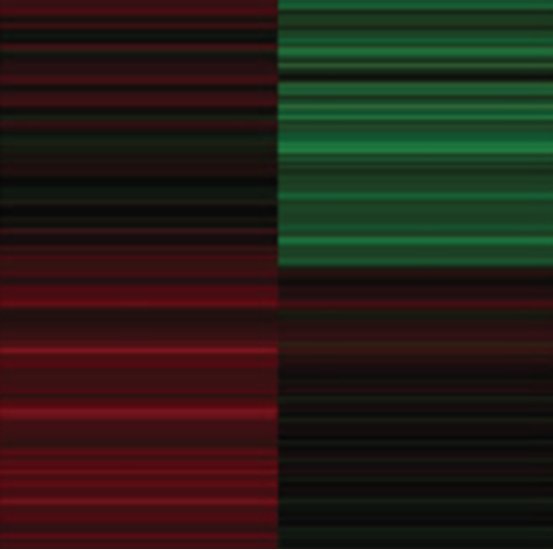

The addition of acetyl groups to lysine residues in proteins is an important step in a wide range of biological processes, including the regulation of gene expression, protein-protein interactions and protein stability (Beltrao et al., 2013; Zhang et al., 2009; Glozak et al., 2005; Kouzarides, 2000). The removal of these acetyl groups by enzymes called lysine deacetylases is also important (Downey and Baetz, 2015).

Two families of lysine deacetylases are known, and they both need a cofactor to be able to work properly (Yang and Seto, 2008). The only lysine deacetylase to have been identified in the bacterium *Escherichia coli* to date is called CobB, and it belongs to the family of enzymes that rely on a chemical called NAD^+^ as a cofactor (AbouElfetouh, et al., 2015). Now, in eLife, a team of researchers from China, Taiwan and the United States – including Shen Tu, Shu-Juan Guo and Chien-Sheng Chen as joint first authors – report that they have used a technique called “clip-chip” to identify a new lysine deacetylase in *E. coli * (Tu et al., 2015).

In general, the clip-chip strategy uses two glass slides: one slide contains thousands of purified proteins, and the other is coated with a protein or molecule of interest. By placing the first slide onto top of the second, one can find out if any of the proteins on the first slide are enzymes that can use the protein of interest on the second slide as their substrate. Tu et al. tested thousands of proteins from *E. coli* against three different substrate slides. The three potential substrates were proteins that are acetylated heavily in *E. coli* cells, but are not deacetylated by CobB. They found that an *E. coli* protein called YcgC was a lysine deacetylase that has RutR – a protein that regulates transcription in *E. coli* – as a substrate. Further experiments revealed that, unlike other lysine deacetylases, YcgC does not require a cofactor. Tu et al. then used mass spectrometry techniques to find two specific lysine residues in RutR that are targeted by YcgC (Figure 1).

**Figure 1.**
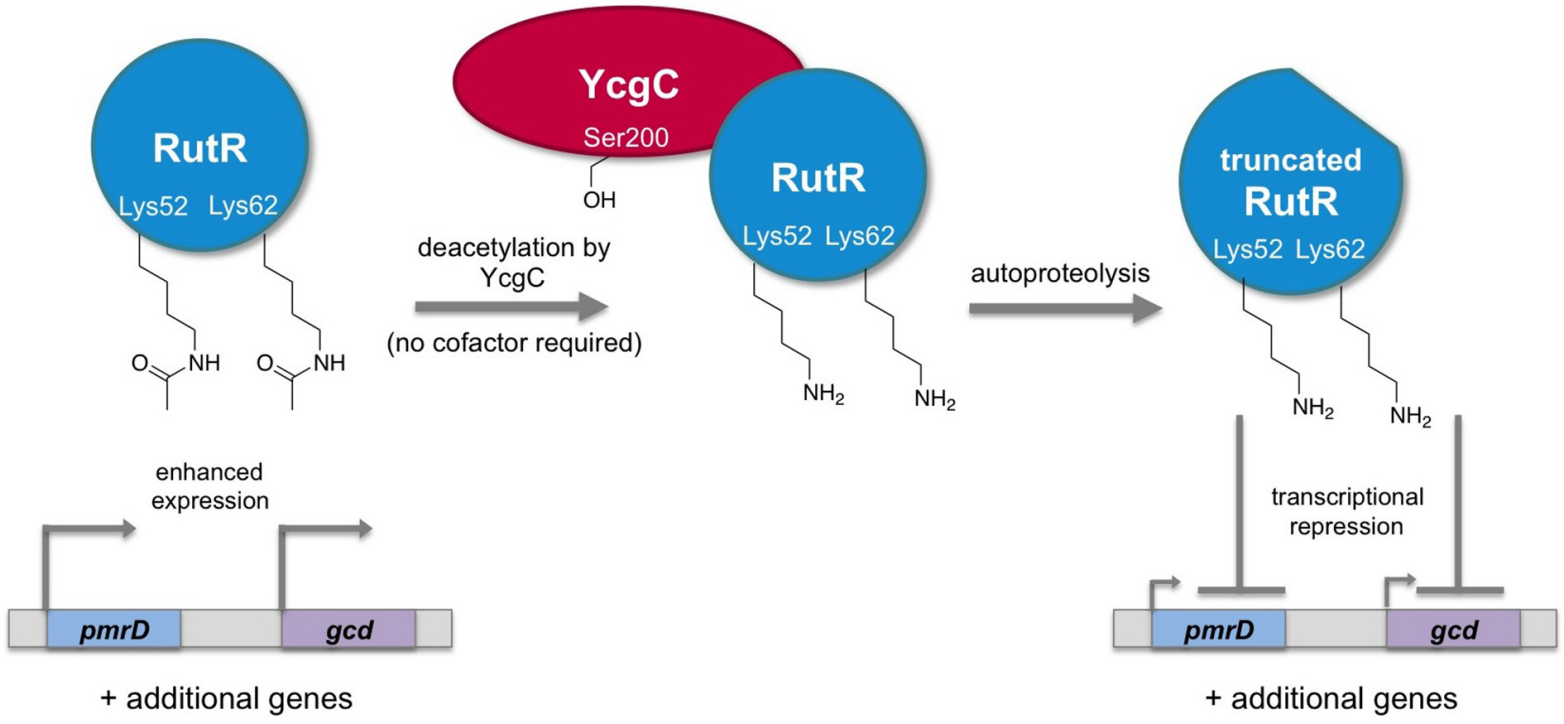
Regulation of gene transcription in *E. coli* by YcgC. The acetylated form of the transcriptional regulator RutR enhances the expression of its target genes, such as *pmrD* and *gcd* (left). Tu et al. have discovered that YcgC can remove acetyl groups (COCH_3_; only the oxygen is shown in the figure) from at least two lysine residues on RutR (Lys52 and Lys62; middle). Moreover, deacetylation of RutR causes it to remove its own N-terminus. This deacetylated and truncated form of RutR represses the expression of *pmrD* and *gcd* (right). Further experiments showed that a serine called Ser200 (sidechain shown with OH) is crucial for YcgC’s catalytic activity.

Deacetylases belong to a broader class of enzymes called hydrolases, which use water molecules to break chemical bonds. By incubating purified YcgC with various chemicals that inhibit hydrolase enzymes, Tu et al. discovered that it belongs to the serine hydrolase family. This was confirmed by replacing the serine residues in YcgC and showing that the mutant enzyme was unable to deacetylate RutR in vitro.

Tu et al. went on to notice that RutR appeared to decrease in molecular weight after incubation with YcgC. Further investigation revealed that this decrease was caused by the removal of several residues from the N-terminal end of the RutR protein. Importantly, this did not occur if RutR proteins were unraveled by heat-treatment, which suggests that deacetylation causes the RutR protein to remove a short section from its N-terminal end in a process called autoproteolysis.

RutR represses gene expression, and further experiments showed that YcgC-mediated deacetylation of RutR led to reduced expression of at least two of its target genes. Further experiments revealed that YcgC regulates a set of substrates that are different to those regulated by CobB.

Tu et al. then searched for other bacterial proteins that looked like they may possess lysine deacetylase activity similar to that of YcgC. Several YcgC homologs were identified in several genera of bacteria, including *Shigella* and *Yersinia*. Furthermore, because YcgC and its homologs do not require NAD^+^ or zinc ions as a cofactor and look different from known bacterial lysine deacetylases, they likely represent a new family of lysine deacetylases.

Finally, we have become accustomed to thinking of lysine acetylation and deacetylation as driving reversible changes in the shape of proteins, with knock-on effects for protein-protein or protein-DNA interactions. However, as demonstrated by the fact that deacetylation leads to the removal of the N-terminus of RutR, this work reveals that they can also be coupled to irreversible protein modifications.

## References

[bib1] AbouElfetouh A, Kuhn ML, Hu LI, Scholle MD, Sorensen DJ, Sahu AK, Becher D, Antelmann H, Mrksich M, Anderson WF, Gibson BW, Schilling B, Wolfe AJ (2015). The *E. coli* sirtuin CobB shows no preference for enzymatic and nonenzymatic lysine acetylation substrate sites. MicrobiologyOpen.

[bib2] Beltrao P, Bork P, Krogan NJ, van Noort V (2013). Evolution and functional cross-talk of protein post-translational modifications. Molecular Systems Biology.

[bib3] Downey M, Baetz K (2015). Building a KATalogue of acetyllysine targeting and function. Briefings in Functional Genomics.

[bib4] Glozak MA, Sengupta N, Zhang X, Seto E (2005). Acetylation and deacetylation of non-histone proteins. Gene.

[bib5] Kouzarides T (2000). Acetylation: a regulatory modification to rival phosphorylation?. The EMBO Journal.

[bib6] Tu S, Guo S-J, Chen C-S, Liu C-X, Jiang H-W, Ge F, Deng J-Y, Zhou Y-M, Czajkowsky D, Li Y, Qi B-R, Ahn Y-H, Cole PA, Zhu H, Tao S-C (2015). YcgC represents a new protein deacetylase family in prokaryotes. eLife.

[bib7] Yang X-J, Seto E (2008). The Rpd3/Hda1 family of lysine deacetylases: from bacteria and yeast to mice and men. Nature Reviews Molecular Cell Biology.

[bib8] Zhang J, Sprung R, Pei J, Tan X, Kim S, Zhu H, Liu C-F, Grishin NV, Zhao Y (2009). Lysine acetylation is a highly abundant and evolutionarily conserved modification in *Escherichia coli*. Molecular & Cellular Proteomics.

